# Seen and Heard: Women and Mother’s Experiences of Navigating a Drug and Alcohol Recovery Community

**DOI:** 10.3390/ijerph23010025

**Published:** 2025-12-23

**Authors:** Lydia Shrimpton, Hayley Alderson, Kim Hall, Monique Lhussier, Ruth McGovern, Zeibeda Sattar, William McGovern

**Affiliations:** 1 School of Communities and Education, Faculty of Health and Wellbeing, Northumbria University, Newcastle Upon Tyne NE7 7XA, UKmonique.lhussier@northumbria.ac.uk (M.L.); zeb.sattar@northumbria.ac.uk (Z.S.);; 2 Population Health Sciences Institute, Faculty of Medical Sciences, Newcastle University, Newcastle Upon Tyne NE2 4AX, UK; hayley.alderson@newcastle.ac.uk (H.A.);

**Keywords:** stigma, recovery, Women Who Use Drugs (WWUD), Mothers Who Use Drugs (MWUD), peer support, drug and alcohol treatment

## Abstract

Women Who Use Drugs (WWUD) are amongst the most stigmatised groups in society and are subject to stigma as they engage with services and within their own recovery communities. WWUD who are also mothers have been found to experience increased stigma and disproportionate surveillance by professionals when accessing services, leading to a constant fear of child removal and apprehension to accessing, engaging and seeking support. In this study, we report findings from a community asset mapping project conducted with drug and alcohol recovery services in the North-East of England. The aim of this study is to examine the gender-specific and recovery-related experiences of WWUD when accessing services and women-only spaces. Semi-structured interviews (*n* = 13) and focus groups (*n* = 4) were carried out with professionals working in the recovery community and women in recovery from substance use. A reflexive thematic analysis approach was used to analyse the data, resulting in three themes being identified: (1) The role of peer support in empowering women in recovery; (2) Navigating recovery as a mother; and (3) Working with women in recovery. Findings revealed that gender-specific groups provide a sense of safety, connection, identification, and empowerment for WWUD. This study further highlights the gender-based stigma WWUD experience when accessing services, and the challenges they experience where appropriate spaces are limited in the recovery community. We conclude by recognising the importance of sisterhood for WWUD and recommending the promotion of gender-specific peer support groups and for practitioners working with WWUD to reflect on their own stigmatising behaviour and how this can manifest in the increased monitoring of women and mothers in recovery.

## 1. Introduction

In drug and alcohol treatment settings, women are largely underrepresented, with the latest statistics from the Office for Health Improvement and Disparities (OHID) revealing that men account for more than two-thirds (68%) of people accessing drug and alcohol services [1]. Of the women accessing treatment for drug and alcohol use, over one quarter (27%) are mothers [1]. The small number of women accessing services for support for substance-related concerns can be attributed in part to the challenges women and mothers experience when in active addiction and recovery, such as acute gender-based stigma and multiple disadvantages, for example, limited childcare provision, social service involvement, sex-related risk behaviours, and domestic abuse [2,3,4,5,6].

Stigma is defined as the devaluing of an individual based on their characteristics and/or behaviours, and it can lead to prejudice and discrimination [7,8]. It is complex and can take multiple forms, such as interpersonal stigma experienced in public and social settings; structural stigma, experienced in healthcare and support service settings; and self-stigma in which the stigmatised individual internalises the negative attitudes, behaviour and labels perpetrated against them [7,9,10]. Women Who Use Drugs (WWUD) have a stigmatised identity [11,12] and experience stigma in all aspects of their lives, including how well they are able to meet the “good mother” ideal, for example, whether they are perceived to be a “good” or “bad” mother by society’s standards [13]. Healthcare professional stigma is a significant concern for WWUD and is reflected in the dismissive and judgmental attitudes of those working with them [14].

Despite an increase in research interest around recovery, there is a dearth of evidence regarding the first-hand experiences of those in recovery, with Mothers Who Use Drugs (MWUD), who are facing and have experienced child removal, often being overlooked in research and the welfare system [2,15,16]. Of the limited literature, existing research has likened service involvement for MWUD to being “under surveillance” due to an increased level of monitoring and strict requirements [12,17]. Women accessing drug and alcohol services are estimated to have had an average of 3.2 pregnancies [18]. Additionally, MWUD are often open to social services and are at a heightened risk of child removal, with them being six times more likely to have their children removed from their custody compared to fathers who use drugs [19]. When exploring barriers to treatment for MWUD, the fear of losing custody of their children has been found to impact their engagement with services, with seeking help largely associated with child removal [15,20,21].

More broadly, WWUD often require different needs to be met compared to their male counterparts [22]. WWUD are at a higher risk of experiencing traumatic and abusive relationships, whilst potentially navigating childcare responsibilities as a sole parent with limited social and family networks to support them [23,24,25]. Because of the strong association between domestic abuse and substance use, women accessing mixed-gender recovery may be doing so alongside those who have or could abuse or exploit them. Moreover, WWUD with a history of abuse are likely to find male-dominated spaces triggering and unsafe [22], and at odds with trauma-informed practice [26]. Trauma-informed practice refers to the recognition of trauma experienced by a person and subsequently adapting practice to prevent triggering and re-traumatisation, whilst supporting the individual. The key components of trauma-informed practice are safety, trust, choice, collaboration, empowerment, and cultural consideration [27]. Thus, WWUD require a trauma-informed approach in recovery settings as they navigate their recovery in the context of trauma, such as potential child removal, abuse, and stigma perpetrated against them [26].

In particular, there is limited literature exploring the benefits of peer support for WWUD and MWUD in women-only spaces as they navigate the balance of recovery, unique multiple disadvantages, and potential childcare. Peer support has been defined as the process of mutually giving and receiving non-clinical assistance from others with similar lived experience [28]. Peer support groups have been evidenced to be a key component of recovery and a secondary intervention alongside treatment, aiding the beginning and maintenance of recovery [28,29,30,31]. Of the existing limited literature, research has highlighted the resilience and comfort women experience when accessing women’s support groups [5].

Therefore, the aim of this study is to examine the gender-specific and recovery-related experiences of WWUD when accessing services and women-only spaces.

## 2. Materials and Methods

### 2.1. Study Design

This study presents findings from a multi-component exploration of Community Asset Mapping (CAM). The research was commissioned by an English local authority and mapped drug and alcohol recovery services across the North-East of England in collaboration with people with lived experience of accessing recovery-oriented services [32,33]. CAM is a strengths-based approach which involves re-engaging and re-developing communities through research, whilst documenting pre-existing resources within the community. In the case of this research, the assets mapped included individuals, services, and community groups, supporting the recovery and wellbeing of people using drugs and/or alcohol. Three work packages were conducted to complete the research and ran consecutively: (1) Public Involvement and Community Engagement (PICE) workshops with local service user and carer forum members to inform the interview topic guides and sense-check findings; (2) Identifying and mapping existing recovery assets in the local community alongside PICE members; and (3) Semi-structured interviews and focus groups with professionals working in the recovery community. Interviews and focus groups investigated perceptions regarding developments of the local recovery community, recovery needs, and recovery provision in the local area. Due to the extensive data collected regarding practitioner perspectives of WWUD’s experiences of accessing recovery spaces, two further focus groups were conducted with women in recovery to explore in depth their first-hand experiences whilst navigating the recovery community. This enabled us to complement and explain further the findings from professionals regarding WWUD’s visibility and experiences in the recovery community.

This study presents findings from the interviews and focus groups with professionals relating to women in the recovery community, and the additional focus groups conducted with women with lived experience of accessing recovery-oriented services. This study used a qualitative research design grounded in social constructionism [34]. Social constructionism enabled us to develop a deeper understanding of the participants’ perceptions, values, and experiences of accessing recovery services and women-only spaces.

### 2.2. Participants and Recruitment

Purposive sampling was used to recruit participants via the research teams’ pre-existing networks with professionals working in the recovery community. Practitioners and service providers were approached by the research team and asked if they would be interested in taking part in a CAM study exploring the recovery community. Professionals as representatives of the Drug Treatment System were also invited to take part if they were working in the recovery community in any capacity. Women in recovery and accessing services for drug and alcohol recovery support were also sought for inclusion in the additional focus groups. Topics explored in the semi-structured interviews and focus groups included: the visibility and inclusivity of the recovery community; individual, social and cultural benefits of a recovery community; the structural, social and individual barriers to developing and accessing a recovery community; how services support recovery and the community; the importance of women-only and mother-only spaces in the recovery community; and experiences of balancing childcare whilst accessing recovery services.

In total, 29 (15 females; 14 males) participants involved in a recovery community in the North-East of England took part in a semi-structured interview (*n* = 13) or focus group (*n* = 4) conducted by LS. Participants were aged 29–60 and were all of white ethnicity. Of the participants, 12 worked in the recovery community with no personal lived experience of recovery; 13 participants identified as having the dual role of being a professional working in the community and being in recovery themselves; and 4 participants identified as in recovery and not working in the community. Participants with lived experience had been in recovery for 6 months and over, however, we did not ask each participant their specific recovery stage to avoid discrimination as advised by our PICE group in our initial study [32,33]. Semi-structured interviews involved 3 females (2 professional; 1 professional with lived experience) and 10 males (4 professional; 6 professional with lived experience). Focus groups were comprised of 16 participants (2 females, 3 males, all professional with lived experience, in focus group 1; 5 females, 1 male, all professional, in focus group 2; 3 females, all with lived experience and 1 also a professional, in focus group 3; and 2 females with lived experience and not working in the community, in focus group 4).

Of the 15 women participating collectively, 6 identified as mothers in recovery. All women in focus groups 3 and 4 identified as mothers in recovery and had experienced social service involvement, with a variation in their experience of child removal.

Interviews and focus groups took place in person (9 interviews; 2 focus groups (focus groups 1 and 2)) and via Microsoft Teams video call software (4 interviews; 2 focus groups (focus groups 3 and 4)), dependent on participant preference. In-person interviews and focus groups took place within participants’ services (*n* = 7 interviews; *n* = 1 focus group), and at a local recovery hub (*n* = 2 interviews; *n* = 1 focus group). It was important for the research team to give the participants a choice regarding the format of the interview and the location to ensure participants were comfortable and to work around professionals’ busy schedules. Interviews and focus groups lasted between 25 and 65 min approximately.

A participant information sheet and a consent form were given to each participant pre-interview, and an opportunity to ask questions was provided. Informed consent was obtained from all participants prior to the interview or focus group taking place.

### 2.3. Analysis

Interviews and focus groups were digitally audio recorded following consent and transcribed verbatim. Transcriptions were anonymised and cleansed of any identifiable data. Field notes taken during data collection were used to support the analysis of transcriptions. Within this study, data saturation was approached using ‘a priori thematic saturation’ [35], with codes from our data with professionals informing what we expected to find when interviewing women with lived experience in our additional focus groups. Whilst we approached the additional focus groups receptive to new codes and themes emerging [36], we considered data saturation to be achieved when our additional focus groups corroborated the findings from our discussions with professionals and did not suggest any data we had not already collected.

Data were analysed following a six-step thematic analysis approach [37]: (1) data familiarisation, (2) code generation, (3) theme generation, (4) theme review, (5) theme definition, and (6) result production. Data were initially analysed by LS, and then WM and HA. Several meetings were undertaken between LS, WM and HA during the data collection and analysis. Meetings were held to discuss emerging codes and any gaps within the data that could be resolved in future interviews, such as the data relating to WWUD. Once all data was collected, a meeting was undertaken to finalise the themes from the agreed codes amongst LS, WM and HA. These were then brought to the wider research team. All research team members were in agreement regarding the codes. The initial themes generated and decided by LS, WM and HA were discussed between the research team to decide whether they accurately represented the data, with codes reorganised until all researchers were in agreement regarding the final codes and themes. These were then taken to our PICE team (see [32,33]), with the session comprising 8 females and 5 males and involving sense-checking our findings. PICE members (*n* = 7 people with lived experience of recovery (5 females, 2 males), *n* = 3 practitioners (1 female, 2 males), and *n* = 3 carers (all female)) discussed the findings in detail, with additional information given corroborating what had already been found. PICE members sense-checked our codes and themes and confirmed that these accurately reflected the data and their experiences.

### 2.4. Positionality

All three authors involved in data analysis (LS, WM, and HA) have a background of working in drug and alcohol recovery and clinical settings and have an extensive research background of exploring substance use treatment. This supported analysis by providing an in-depth knowledge of how recovery services operate and the impact this has on women from a professional perspective. The wider research team is varied in personal experience and expertise, with the majority of members having a background of working in practice, including recovery and clinical settings, and social services. All research team members have an extensive background in research, looking at multiple disadvantages in marginalised communities. This benefited the research as the team were able to collate their knowledge from their research backgrounds and their experience of working in multiple different recovery-based settings when interpreting the data. The majority of the research team also have parental responsibilities with this providing a deeper level of understanding when analysing findings relating to navigating childcare and service provision. The researcher’s (LS) positionality when conducting the interviews and focus groups was as a female with previous experience working in criminal justice and drug and alcohol recovery settings. This enabled LS to provide a deeper level of understanding and an empathetic approach to the fieldwork and created a comfortable space in which participants could discuss their experiences using acronyms, allowing for conversations to flow more easily. LS has a background of extensive training to work with people with multiple disadvantages, such as substance use, allowing her to approach the interviews and focus groups carefully and sensitively, and monitor participants’ comfort levels and any concerns. LS engaged in reflexive writing when documenting her field notes to mitigate any bias and had regular meetings with WM following interviews and focus groups to discuss the interview content and emerging themes. This ensured that any personal biases and assumptions of LS were being managed.

## 3. Results

The following key themes emerged from the qualitative data and are outlined below: (1) The role of peer support in empowering women in recovery; (2) Navigating recovery as a mother; and (3) Working with women in recovery.

### 3.1. The Role of Peer Support in Empowering Women in Recovery

Professionals discussed how women in the recovery community were less visible than their male counterparts, with this partially attributed to women- and mother-only spaces being scarce in the recovery community. Participants expanded on this observation further by stating how women and mothers in recovery were creating and leading their own groups to fill an unmet need, as opposed to provision being found in existing commissioned services. Due to the limited visibility of women-only spaces, participants felt that support is not overtly promoted, instead WWUD are often left to rely on a “word of mouth” approach to discover what gender-specific services are available to them:

*“I just feel like there is not much information out there about things […] And I feel like if I had been introduced to women’s spaces and wellbeing stuff and stuff like that earlier on, maybe my life would have been a bit different.”* (Participant 4, Female, Lived Experience)

The quotation above demonstrates how women in recovery discover women-only spaces once they are already embedded in the recovery community, with the knowledge of these spaces dependent on who they encounter in their recovery journey. This can result in women not accessing women-only spaces for over a year into their recovery, with inequitable access to support dependent on how immersed each individual is in the recovery community. This suggests a need for more visible recovery services to make recovery a more achievable goal to strive for. Women in recovery highlighted the perceived importance of finding women-only spaces early; this was particularly true for women who had negative experiences with men prior to and during their recovery. There was a shared agreement that women-only spaces provided a sense of safety in contrast to mixed-gender spaces, where women sometimes felt vulnerable and triggered by past traumatic experiences, which impacted their ability to fully engage in recovery work:

*“Women have different needs and wants, and different histories from what men do”* (Participant 6, Male, Service Provider)

Women with lived experience reflected on their experiences of mixed-gender recovery settings and how male-dominated peer support groups hindered women from feeling able to talk about the reality of active addiction and being a mother without fear of judgement. Male-dominated spaces and mixed gender groups also failed to recognise the nuanced needs of WWUD or respond to the potential power dynamics present between male and female drug users and how this can directly influence levels of perceived/actual risk and impact on the overall effectiveness of available interventions and/or support.

There was a general consensus amongst women in recovery that women- and mother-only peer support spaces were able to overcome the isolating experience of stigma associated with being a woman or mother who uses substances. Mothers in recovery described mother-only peer support groups in particular as a “sisterhood” and highlighted their ability to provide for mothers the core components of CHIME: a sense of connectedness, hope, identity, meaning and empowerment [38]:

*“One of the things that really helped me in my recovery was my little boy, who’s 10 now, he was a baby when I first got into recovery and meeting with other mams […] I could be the person that I needed to be, to be able to recover”* (Participant 7, Female, Service Provider with Lived Experience)

Mothers reflected on peer support, further stating that it helped provide validation *“that you’re okay and that you’re not a bad mam”* (participant 5, Female, Live Experience). Mothers commented on how peer support with other mothers in recovery also helped provide their children with an opportunity to engage with other children experiencing parental substance use. This, in turn, had potential for their children to recognise they had shared experiences and were not alone:

*“They all brought their children along as well, and my kids have been different with me ever since that day […]‘cos they now know they’re not alone. They’re not the only child in the world who’s got a mam who’s like me.”* (Participant 5, Female, Lived Experience)

Overall, this theme reveals how peer support can be vital to a woman in recovery in providing a sense of safety and identification with other women. Social capital refers to positive resources stemming from an individual’s participation and relationships within their recovery community that benefit their recovery [39], with our findings demonstrating how peer support can encourage social capital for women in recovery.

### 3.2. Navigating Recovery as a Mother

Participants who had experienced child removal or social service involvement due to substance use and had regained custody of their children during the recovery revealed their fear of seeking help from services. Mothers equated seeking help with a guarantee that services will *“take our children off us”* (Participant 2, Female, Lived Experience). Asking for help was associated with feeling as though they were being “punished” as they risk their child being removed as a consequence:

*“I didn’t know how to parent. I didn’t know how to be a mam and I wanted to access help for that, but I was scared that they were going to take her off me […] I felt like I couldn’t say, “listen, I’m struggling” because I didn’t want to be judged and I didn’t want her being removed again from us.”* (Participant 4, Female, Lived Experience)

Mothers who had also been in this position of regaining custody of their children reflected on the importance of peer support during the initial stages of parenting again. They described how other mothers were able to share their experiences, provide support, and teach them about parenting without the fear of negative repercussions as they transitioned from a child being removed to having them returned to their care. Mothers in recovery discussed how they felt services were not equipped to support them with their parenting role. Furthermore, some mothers stated that they felt like services did not have the capacity to work with them holistically regarding their needs and were only able to provide care regarding their substance use. Mothers reflected on service provision further, noting how a lack of child-friendly spaces and creches in recovery settings often made it challenging for mothers to protect their children from being exposed to intoxication, undesirable behaviour, and/or conversations around substance use:

*“My other children, being younger [were] sitting in the waiting room, with a lot of other, like, people who were talking about […] who’s selling what and who’s doing this… “I’ve been taking this; I’ve had some of that”. I found that really difficult, like, especially when you’re trying to protect your children from that stuff”* (Participant 2, Female, Lived Experience).

A small number of professionals working in the community commented on the limited amount of known creches across recovery services, recognising how services were not adapting to the needs of mothers and posing an extra barrier for service engagement. Participants highlighted that not all mothers in recovery have family networks who are able to care for their children whilst they attend appointments. They reflected on this further, commenting that this delayed the ability to seek recovery support. Participants noted that this can be particularly challenging for women who have relocated for their safety from domestic abuse:

*“There’s women in there that are being protected, basically, from domestic abuse, but they come in and they’re using […] They’re not allowed to engage with the services, whilst taking children with them. So basically, they have got to wait until their children have been allocated to a new school before they’re allowed to engage with the services, and then, I’ve known, like, a couple of women now to have lost their children, because they haven’t been engaging with services”* (Participant 1, Female, Lived Experience)

As seen in the above quotation, participants felt that the limited childcare provision created an impasse in which mothers were unable to attend services due to limited options for childcare, which then led to child removal due to their limited engagement with services. Participants who had experienced social service involvement during their recovery, but still had custody of their children, discussed a feeling of guilt when accessing services with mothers who had experienced child removal. They recognised the re-traumatisation that this could cause for others:

*“Sometimes they will let us take my daughter along [to a peer support group for mothers], but then I will feel guilty, because there are women there who cannot see their children and there is me with my daughters […] I feel like they are more entitled to the groups than I am because I have got custody of my children”* (Participant 5, Female, Lived Experience)

As seen above, mothers in recovery felt they were unable to bring their children to meetings despite the limited creche options available, as they were concerned about their children triggering other mothers who had experienced child removal. Where mothers have accessed recovery settings with their children, participants described a stigmatising and “humiliating” experience due to their children not having a space, therefore, being present for all aspects of their recovery:

*“I can remember having to take my kids [to an appointment] […] I can remember how humiliating that was […] they used to just hand me a pot to go and wee in. “Mam’s going to wee in a pot” […] Some staff were very lovely […] But then you had like other staff who were like, “you’re on a script; you’re scum. Piss in this.”* (Participant 2, Female, Lived Experience)

Mothers further commented on feeling as if they were stripped of any privacy or confidentiality as they navigated motherhood whilst being open to services. Women described scenarios within which they had decided to share information in the hope of receiving the necessary support; however, they experienced negative consequences, such as those shared by participant 4 below:

*“When she [daughter] was 11 and she was getting removed again, I told the social worker that it was really bad and that I wanted to leave my partner and they basically announced it in a social services meeting while she [daughter] was there and I had to deny it.”* (Participant 4, Female, Lived Experience)

This sense of betrayal after sharing in confidence with a practitioner resulted in a feeling of fear to share again in the future. There was a shared agreement amongst participants with lived experience of social services involvement that mothers felt unable to recover privately when open to social services and were craving a safe space where they were able to discuss their recovery with other women without fearing the negative repercussions of this. As seen above, these participants felt the breach of privacy and consequences they had experienced in the past when sharing in confidentiality had increased the risk they were exposed to and led them to feel unable to share again. Participants continued by describing how, when open to social services, they felt unable to achieve goals set for them to maintain custody of their children due to unrealistic goals being set:

*“Your child is removed for mental health issues, there’s an eight-month waiting list to get your mental health sorted, like, to see a counsellor, but you’ve only got 26 weeks to sort your life out […] I felt like I was left to die”* (Participant 4, Female, Lived Experience)

As seen in the above quotation, women with lived experience felt that the timescales given to them by services are impossible to meet, and mothers can feel neglected by services in the process. Whilst the focus on the child was recognised as paramount, it often felt that the mother and child’s needs were seen as independent entities, rather than intertwined realities. The mothers further explained that they felt that they were never able to meet the standards expected for them from social services, and that their child(ren) were used as the reward at the end, although services kept “moving the goalpost”. This led to feelings of demotivation and disempowerment:

*“You’re expected to jump through these hoops and then they’ll give you a goal, you’ll get to that goal and then the goalpost moves again […] Theres already barriers there but they put more barriers up and it doesn’t matter what you do at times […] when they’re moving the goalposts and you get so far and you think: right, I’m nearly there, I’m nearly there, I’m going to get my kids back”* (Participant 3, Female, Community Worker with Lived Experience)

This perception of service goals being impossible to achieve can lead to mothers feeling they are constantly trying to convince services that they meet the “good mother ideal” [13], and to *“prove myself that I wasn’t a bad person, I was not a bad mother”* (Participant 3, Female, Community Worker with Lived Experience). Participants discussed how the responses of services can lead them to stigmatise themselves as they encounter unattainable goals by services and/or feel judged by the professionals who should be in roles to support them.

Overall, this theme highlights the challenges mothers in recovery experience whilst balancing childcare for their children and engaging with services for recovery support. Further, it demonstrates the pressure mothers experience when working towards achieving the goals set by services, with custody of their children seen as at risk if they fall short of services’ expectations.

### 3.3. Working with Women and in Recovery

Participants with lived experience described accessing and being open to services as a stigmatising experience, with the level of stigma they encounter dependent on the worker they are assigned to. Participants felt that a worker who was not stigmatising and understanding was a novelty, with participants often feeling they were under constant surveillance when open to services during their recovery:

*“[social workers] were coming in my house; they were checking my fridge; they were checking my bins; they were checking my toilet […] This is before my drug use spiralled. That was just because I was in a domestic violence relationship […] [I was] under surveillance, massively under surveillance.”* (Participant 5, Female, Lived Experience)

As seen in the above quotation, surveillance from services can feel *“violating”* (Participant 5, Female, Lived Experience), especially where women have past experiences of abuse in which they have already suffered a level of surveillance and control. When discussing service involvement further, participants explained that they felt support had not improved for women over the years. Practitioners recognised the impact of high staff turnover in services, with this requiring women to share their traumatic experiences multiple times throughout their recovery and engagement in a service. When reflecting on child safeguarding in services, participants agreed that there was a need for safeguarding to be conducted in collaboration with the mother:

*“Prior warning. Someone sitting you down and saying, “listen, this is what we’re going to do; we’re going to put a safeguarding issue in, but we are also going to signpost you to this, this and this, and help you. We’re not going to leave you alone. We will advocate for you if needed…” I feel like it’s really important to treat women like human beings [and that] they know exactly what’s going to be happening and then we’re prepared”* (Participant 4, Female, Lived Experience)

Mothers with lived experience in this study acknowledged that they had needed social service support with their children; however, they remarked on the impact this had when processes had been undertaken and safeguarding referrals had been completed without their knowledge; this left them feeling at a disadvantage. The use of unexplained jargon in child protection meetings was also noted as isolating and excluding to mothers, with mothers with lived experience reporting how this meant they were unable to contribute to conversations regarding their children. This left them with a sense of not feeling seen or heard and suggests missed opportunities to recognise behaviours that were a resource for the child, alongside recognising the risk factors present.

Mothers described structural stigma in non-recovery spaces, also, with them feeling like they received less support as a MWUD compared to other mothers. When discussing stigma experienced in hospital settings, mothers described feeling that blame was immediately attributed to them before medical investigations when their children were unwell. Mothers commented on how structural stigma can lead them to avoid seeking medical attention when needed, as they feel there is a negative halo effect for MWUD. Participants commented on how the title of “drug addict” is enduring regardless of the stage of recovery a person is in, with this also recognised by professionals working in the recovery community. Women with lived experience explained further how the persistent nature of stigma can lead to discrimination in all aspects of their lives:

*“D’you know if you’re trying to apply for housing or for a driving license, or even us going to the dentist, I have to declare that I used to be addicted to drugs. So that is constantly following me. What you don’t have to declare though is the fact that I’m nearly five year free from heroin. You don’t have to declare that. So, this title of being a drug addict follows me around no matter where I go. So, it’s constant discrimination”* (Participant 5, Female, Lived Experience)

This theme highlights the need for WWUD to have support, advocacy and guidance from services whilst navigating recovery. It further suggests that WWUD experience stigma in all aspects of their recovery and life, with this being enduring regardless of how long they have been in recovery.

## 4. Discussion

Women-only spaces and peer support groups are able to provide a sense of safety for women and encourage an uplifting “sisterhood” approach to recovery. The concept of a “sisterhood” in recovery refers to the ways in which WWUD and MWUD can support each other through mentorship, collaboration, and mutual encouragement to overcome systemic barriers and stigma. It further provides a social space to celebrate success, advocate for one another, and build authentic relationships with peers in an inclusive and equitable way [40]. Thus, our findings argue that there is a fundamental need to provide a sense of social and psychological safety between women as they navigate recovery and the unique stigmatising experiences associated with being a WWUD and/or a MWUD [5]. Peer support has been found to aid WWUD’s recovery whilst providing a sense of connectedness, hope, identity, meaning and empowerment, with these components found to be key in the progression and maintenance of recovery [5,38]. Identification with other women, particularly for MWUD, can help to combat the structural stigma and subsequent self-stigma experienced as MWUD battle the label of being a “bad mother” [13]. The stigma that mothers face as active drug users in relation to their personality and characteristics does not end when mothers stop using drugs [14]. Most recent research has shown that stigma continues for mothers in recovery and that stigma can actually worsen as mothers become more aware of stigma and have to negotiate and access recovery services [12,14]. Social constructionism is a theory that emphasises the importance of social interactions and, in this case, how these influence a WWUD regarding how they construct their social reality [34]. Thus, theoretically viewed through a social constructionism lens, our findings demonstrated how the ongoing and continued access and nature of peer support is particularly important where stigma was found to be enduring as a social reality and seen in all aspects of the women’s lives, such as in medical appointments and housing applications, regardless of what stage of recovery a woman is in. We argue that MWUD benefit specifically from peer support with other mothers and need this specific intervention, where they are able to advise and support each other in parenting, as an alternative to more formal routes of parenting support that feel inaccessible for MWUD. Peer support for WWUD in general enables women in recovery to rely on each other in an open and non-judgmental space when needed, without being shamed or disproportionately held to account.

Women in recovery experience unique challenges when accessing services, such as balancing childcare where they are the primary caregiver, and gender-based stigma, with theseoften leaving women to feel they fall short and cannot reach the impossible demands of them [3,5,6]. The feeling of unattainable goals being set and constantly being out of reach is particularly felt by MUWD, with previous findings revealing how services were perceived as focusing on the deficits and not the strengths of parents in recovery [41,42]. Our findings and previous literature have evidenced how WWUD can experience a negative halo effect in which negative attributes and stigmatising beliefs are assigned to them in healthcare settings because it is known that they use drugs [43]. This is corroborated by previous research [14] and demonstrates a need for services to engage in anti-stigma training to avoid a non-stigmatising practitioner being described as a novelty by women in recovery. Consistent with previous literature, our findings demonstrated that a fear of child removal is a significant barrier to help-seeking for MWUD, with mothers disclosing that accessing services for support is associated with being “punished” and has consequences in the form of child removal [15,20,21]. This policing by MWUD of assessing whether help-seeking will lead to punishment can be explained by Foucault’s concept of Panopticism, in which the MWUD feels constant surveillance from services and, in turn, polices and self-stigmatise themselves as being “bad mothers” [44]. MWUD require a space where they are able to recover privately without being under surveillance from services [45]. Although there is a need for safeguarding and social service involvement in a family unit where there is active addiction, we argue that safeguarding should be performed in collaboration with the MWUD where possible and appropriate, with women often isolated and excluded from decision-making, removing their voice and an opportunity to be “seen and heard”.

Our discussions with professionals evidenced that women-only spaces are scarce in the recovery community, with existing groups not widely promoted and predominantly found via a “word of mouth” approach. The lack of provision and visibility for women-only spaces in the recovery community demonstrates a need for improvement in recovery settings to promote and provide alternatives for women to navigate recovery without needing to access male-dominated recovery spaces first. Literature has argued that policies and services labelled as gender neutral are in reality male-based, therefore challenging for women to fit and adapt into these spaces [46]. Spaces designed for and specific to women are particularly important for women who have experienced trauma such as domestic abuse and child sexual abuse by male perpetrators, placing them at a heightened risk of being exploited and engaging in survival sex as they access mixed-gender recovery spaces [47,48]. Without women-only spaces, these women are asked to access recovery alongside their male counterparts and potential abusers, thus affecting their feeling of safety whilst navigating recovery [22]. There is a need for trauma-informed approaches to be taken when working with WWUD, with WWUD requiring practitioners to recognise their lived experiences and how previous traumas are accumulative and can impact their capacity to access support [27]. The literature and our findings highlight the importance of these spaces for WWUD, with past experiences of trauma leading them to approach mixed-gender recovery spaces with fear and often a vulnerability [22].

### Strengths and Limitations

A strength of this study was the research team’s pre-existing professional relationships with a large proportion of our sample, with this creating a space in which participants were already comfortable with the researcher and felt able to share their observations and experiences in an open and honest way. To avoid recruitment bias, professionals not already known to us were also recruited, and the research team were intentional in not only recruiting those who were like-minded. Regarding our sample, it is worth recognising that the focus groups with WWUD were conducted as a result of emergent findings on the provision for WWUD within the recovery community from a professional perspective. Therefore, female professionals with a dual role of lived experience and working in the community, who took part in the initial interviews and focus groups, were not asked exclusively about their experiences of accessing the community but their experience of working within the community and observations regarding its visibility and inclusivity. Although our initial interviews with professionals did prompt conversations around women in recovery and resulted in personal experiences being shared from women with lived experience, the main breadth of our data regarding first-hand perspectives of accessing recovery communities was collected from the additional focus groups. Although our recruitment stopped due to achieving data saturation, we recognise that our findings from our sample of women with lived experience mostly reflect findings from women who have similar characteristics of being open to services and in recovery. To resolve this, we asked women in the interviews to discuss their experiences of active addiction and recovery prior to service involvement; however, future research could explore in depth the experiences of women whilst in varying stages of addiction and service involvement.

Due to our initial study exploring the recovery community via community asset mapping, demographic information for female participants was also not collected regarding how many had experienced child removal; therefore, this was not asked of our additional focus group participants. As such, we were unable to compare stages of social service involvement or child removal with experiences of accessing treatment when discussing our findings for this study. Another limitation of this study is that our participants were all living in the North-East and identified as White-British. Due to this, we were not able to discuss the benefits and barriers to peer support for those belonging to other ethnicities and cultures. Therefore, future research could explore the perspectives of women-only spaces for those with additional marginalised factors, such as ethnicity, and explore what provision is available nationally for WWUD.

## 5. Conclusions

Overall, our findings were able to demonstrate the challenges WWUD experience when accessing recovery, such as gender-based stigma and limited appropriate spaces for women in recovery to feel a sense of psychological safety and service understanding of the unique experience of being a WWUD. Thus, our findings highlighted the benefits of peer support for women in recovery, with peer support in gender-specific spaces allowing women in recovery to find identification, empowerment, a sense of safety, and subsequently combat self-stigma. Despite this, women-only spaces were described as challenging to find, with knowledge of spaces often requiring the WWUD to already be firmly integrated into the recovery community. We recommend that women- and mother-only spaces be promoted in existing recovery settings to ensure all women and mothers in recovery are aware of what options are available to them. It was evident in our findings that compassion is lacking towards MWUD when open to services, with mothers often neglected in the mother-baby dyad, particularly when children’s social services are involved. The stigma highlighted in treatment and healthcare settings suggests a need for practitioners to reflect on their own gender-based stigma and how this can manifest into a disproportionate and increased monitoring of MWUD.

## Data Availability

Data are available from the author upon reasonable request.

## Abbreviations

The following abbreviations are used in this manuscript:

WWUD	Women Who Use Drugs
MWUD	Mothers Who Use Drugs
CAM	Community Asset Mapping
